# Potential Metabolic Biomarkers in Adult Asthmatics

**DOI:** 10.3390/metabo11070430

**Published:** 2021-06-30

**Authors:** Soyoon Sim, Youngwoo Choi, Hae-Sim Park

**Affiliations:** Department of Allergy and Clinical Immunology, Ajou University School of Medicine, 164, Worldcup-ro, Yeongtong-gu, Suwon 16499, Korea; simm27@ajou.ac.kr (S.S.); cyw3789@aumc.kr (Y.C.)

**Keywords:** asthma, biomarker, diagnosis, inflammation, lipid, metabolite, treatment

## Abstract

Asthma is the most common chronic airway inflammation, with multiple phenotypes caused by complicated interactions of genetic, epigenetic, and environmental factors. To date, various determinants have been suggested for asthma pathogenesis by a new technology termed omics, including genomics, transcriptomics, proteomics, and metabolomics. In particular, the systematic analysis of all metabolites in a biological system, such as carbohydrates, amino acids, and lipids, has helped identify a novel pathway related to complex diseases. These metabolites are involved in the regulation of hypermethylation, response to hypoxia, and immune reactions in the pathogenesis of asthma. Among them, lipid metabolism has been suggested to be related to lung dysfunction in mild-to-moderate asthma. Sphingolipid metabolites are an important mediator contributing to airway inflammation in obese asthma and aspirin-exacerbated respiratory disease. Although how these molecular variants impact the disease has not been completely determined, identification of new causative factors may possibly lead to more-personalized and precise pathway-specific approaches for better diagnosis and treatment of asthma. In this review, perspectives of metabolites related to asthma and clinical implications have been highlighted according to various phenotypes.

## 1. Introduction

Asthma is characterized by chronic airway inflammation with complex interactions among genetic, epigenetic, and environmental factors [1,2], resulting in constant attempts to understand asthma pathogenesis underlying each phenotype. Although the classification of asthma phenotypes as eosinophilic, non-eosinophilic, or paucigranulocytic phenotypes has enabled intensive treatment and improved clinical outcomes [3], attempts to develop novel approaches are needed to unveil the pathogenic mechanisms of asthma. Since the emergence of new technologies, “omics,” their application in medical research has been rapidly growing [4]. In particular, metabolomics is one of the latest approaches to detect metabolites for phenotype categorization and biomarker discovery in diverse diseases [5]. Metabolites are the intermediate or end products of cellular metabolism required for the maintenance of biological homeostasis and normal cell function [6]. Therefore, alteration in metabolites reflects physiological or pathological states [7]. With the integration of multiple omics, metabolomics is becoming a more powerful tool in clinical application for early diagnosis, prognosis, and treatment of diseases [8]. Despite an increase in metabolomic studies on asthma, it is not clear how metabolites act as key determinants for each phenotype [9]. This review highlights recent progress in the genomic, transcriptomic, proteomic, and metabolomic signatures involved in the pathogenesis of asthma, suggesting the potential for applying this approach in precision medicine.

## 2. Risk Factors in Asthma Pathogenesis

### 2.1. Genetic and Epigenetic Factors

Asthma pathogenesis is still poorly understood, as the airways are influenced by multiple environmental and genetic factors. Accumulating evidence has supported the genetic traits of asthma [10,11], suggesting specific genes involved in some phenotypes of asthma. The conventional studies on asthma-related genes were performed with linkage analysis and positional cloning. More recently, since the first introduction of genomic-wide association studies (GWAS) in asthma [12], multiple genetic variants were validated to explore disease-related regions showing differences in DNA sequences between normal and asthma groups [13]. These findings brought a huge improvement to asthma genetics by identifying novel genetic factors for asthma. However, the gene-gene interaction still remains a problem to solve in genomic analysis [14]. In addition, epigenetic studies have been highlighted focusing on genomic adaption to environmental stimuli, such as DNA methylation, histone modification, or non-coding RNAs, which are not caused by alterations in DNA sequences [15]. With the application of epigenome-wide association studies (EWAS) in asthma, DNA methylation has been suggested to mediate gene expression in immune cells, including T cell polarization [16] or monocyte differentiation [17]. In particular, methylation of cytosine at the carbon-5 position in CpG dinucleotides is regarded as a key factor for initiating a transcription and regulating cell-specific genes in inflammatory diseases [18].

### 2.2. Environmental Factors

Environmental exposure could impact a rapid increase in the prevalence of asthma in populations with a similar genetic background where allergen exposure is a crucial risk factor for asthma [19]. Sensitization to allergens, such as dust mites and animal dander, is related to Th2 immune response with IgE production and eosinophilia, contributing to the development and exacerbation of allergic asthma [20,21]. Other environmental factors, including smoking, air or occupational pollutants, and virus or bacterial infection, are involved in innate immunity and trigger asthma exacerbation [22]. Air pollutants, including particulate matter, nitrogen dioxide, and sulfur dioxide, are known to aggravate inflammatory response related to oxidative stress and injury in the airways, driving enhanced risk of sensitization to inhaled allergens and symptom exacerbation [23,24].

## 3. Identification of “Omics” Markers in Asthma

### 3.1. Genomics

Numerous genetic studies have revealed that multiple genes are involved in the development and progress of asthma by linkage analysis and association studies. In particular, linkage analysis with positional cloning identified diverse genetic markers related to asthma in chromosomal loci [25,26,27,28,29,30,31,32]. In addition, candidate genes association studies identified asthma-related traits, focusing on different allele frequencies of a single nucleotide polymorphism in case-control studies [33]. Since the application of GWAS to asthma, large meta-analyses in European [34] and American cohorts [35] have identified variable genetic determinants (Table 1). Nevertheless, GWAS have the limitations of a small sample population and lack of significant associations [36]. Moreover, the susceptibility of genetic variants for asthma was not replicated across all relevant studies [34].

### 3.2. Transcriptomics

Transcriptomics quantifies transcripts in organisms to identify differentially expressed genes under specific conditions. The main analyzing tools for transcriptomics include microarrays and RNA sequencing [147]. Transcriptomics is usually carried out in blood because of its great efficiency and convenience for analyzing gene expression [148]. Some studies have performed transcriptomic analysis in blood [149,150], bronchial tissue [151,152,153,154], sputum [155,156,157,158,159], nasal brushings [160], bronchoalveolar lavage fluid (BALF) [161], and mixed samples [162], facilitating differential phenotypes in asthma. Finally, recent studies found 90 novel genetic classifiers for asthma, which could be utilized as potential biomarkers [160]. In addition, several studies have investigated distinct gene profiles to determine the severity of asthma (Table 2).

### 3.3. Proteomics

Proteomic studies include protein identification, quantification or localization, post-translational modification, and protein-protein interactions [163]. Liquid chromatography is a typical analyzing tool for proteomics with coupling of mass spectrometry (MS) [164]. Several studies have performed proteomic analysis and identified different protein profiles of asthmatics in serum [165], sputum [166,167], BALF [168,169,170,171], or bronchial biopsy [172] (Table 3). Commonly, these proteins have been found to be involved in multiple biological processes, such as immune response, defense response, lipid metabolism, molecular transport, cell adhesion, and complement activation [166,168,172].

### 3.4. Metabolomics

Metabolomics is an analysis of low molecular compounds involved in biological processes [6]. For metabolic profiling, MS has been widely employed because of its simplicity and sensitivity [173]. Through its combination with chromatography, MS has become a more powerful tool with high resolution and accuracy, extending its application across biological and medical fields [174]. In addition, nuclear magnetic resonance (NMR) spectroscopy was introduced for an alternative analytic tool, using the differences in the direction and speed of nuclear spin on the magnetic field [175]. Metabolic profiling could be performed in various biospecimens according to its purpose (Table 4). Most studies have used blood [176,177,178,179,180,181,182,183,184], urine [185,186,187], or sputum samples [184], since they are easy to collect and are reflective of whole body metabolism [188]. A few studies have analyzed exhaled breath condensate [189,190,191,192,193] and BALF because of their relevance to the airway physiology [194].

### 3.5. Limitation of “Omics” in Biomaker Identification for Asthma

“Omics” enabled the comprehensive evaluation of holistic molecules in an organism. This advantage leads to the advances in biomarker discovery in medical fields [195]. Genomics is the first study of the omics and aims to search for genetic variants associated with specific diseases [4]. Although it has provided a comprehensive understanding for asthma, it could not explain a mild to moderate asthmatic state [196]. Moreover, the number of samples or replication tests was not sufficient [33]. In contrast, transcriptomics focuses on the differential gene expression and activity of functional genes in patients [197]. Nevertheless, DNA or mRNA expression may not represent the biological function of genes at the protein levels due to translational regulation or post-translational modification [198]. Proteomics reflects pathological changes at the time because cytokines or chemokines are important protein related to the severity of inflammatory diseases [199]. In asthma, transcriptomics and proteomics could be applicable to a wide range of cell types and biological samples including blood, BALF, sputum, and bronchial or nasal brushings [200]. However, they could not provide overall information underlying diseases due to a large variability in sample types or disease states [201]. To overcome these limitations, metabolomics was suggested as one of the latest omics technologies. In particular, its application is rapidly increasing in asthma for the detection of volatile organic compounds in EBC with a noninvasive way [202]. However, the lack of replication or standards of metabolic profiling in different biospecimens is still challenging [9]. Despite the outstanding achievements of omics studies, they are still considered insufficient to mainly be utilized in clinical practices. Therefore, the integration of multiple omics could complement the limitation of each technology.

## 4. Metabolic Pathways Involved in Asthma

### 4.1. Amino Acid Metabolism

Amino acids are involved in multiple biological functions including growth, maintenance or development, regulation of gene expression/cell signaling, and synthesis of nitrogen/protein/energy substrates for homeostasis in organisms [203]. Moreover, they are known as crucial regulators of metabolic pathways for immune responses and anti-oxidant activities [204]. In particular, arginine has been suggested to affect the development of asthma. It synthesizes nitric oxide (NO) by NO synthase (NOS) composed of 3 isoforms, neuronal NOS (nNOS), inducible NOS (iNOS), and endothelial NOS (eNOS) [205]. In asthmatic patients, expression of iNOS and arginase-1/2 were upregulated in the serum or epithelium and immune cells of the airways, leading to higher levels of exhaled NO and more severe symptoms [206,207,208]. Furthermore, arginase metabolism is involved in the regulation of T-cell function, driving Th2 airway inflammation [208]. However, suppressive roles of arginase-2 have been reported in severe eosinophilic and neutrophilic inflammation in asthma [209]. In addition to arginine, higher levels of β-alanine [185] and lysine [190] were detected in patients with severe asthma. However, another study showed conflicting results with decreased levels of arginine, alanine, leucine, valine, and histidine in asthmatics [177]. Therefore, further studies on various phenotypes of asthma are needed to clarify with this pathway.

### 4.2. Lipid Metabolism

The importance of lipid mediators in respiratory diseases is highlighted due to their involvement in various biological functions, such as cell structural components, energy sources/metabolism, signal transduction, and material transport [210]. Among several types of lipids, fatty acids are known to be relevant to diverse mechanisms of inflammatory response in chronic airway diseases [211]. Moreover, arachidonic acids have been identified to contribute to asthma pathogenesis as precursors of eicosanoids including leukotrienes, prostaglandins, thromboxane, lipoxins, and hydroxyeicosatetraenoic acids [212]. In particular, urinary leukotriene E_4_ (LTE_4_) has been widely used for a diagnosis of a distinct phenotype of severe eosinophilic asthma, referred as aspirin-exacerbated respiratory disease (AERD) [213]. In addition to fatty acids, several studies reported that sphingosine-1-phosphate (S1P) levels were increased in asthmatics and correlated with asthma severity [214,215,216]. Furthermore, increased levels of S1P (derived from various inflammatory cells) were found in patients with AERD [217]. Despite the controversial functions of lipids, it is certain that they are key mediators in asthma pathogenesis. Therefore, lipids might be potent biomarkers for specific phenotypes of asthma and promising candidates for new therapeutic targets.

## 5. Changes in Metabolite Profiles According to the Phenotype of Asthma

### 5.1. Mild-to-Moderate Asthma

Asthma has been classified as mild, moderate, or severe types according to the severity of symptoms and frequency of asthma exacerbation. Mild-to-moderate asthma can be defined as a controlled one by step 1-4 treatments [218]. Several studies have performed metabolic analyses to identify metabolic determinants related to the severity of asthma. As a result, increased 8 (α-linolenic acid, linoleic acid, oleic acid, linoleoyl ethanolamide, dodecanedioic acid, linoleates, methylcysteine, and theobromine) and decreased 6 metabolites (indole-3-acetate, lysine, lyso-platelet activating factor C16:0, methionine, phenylalanine, and phenylacetyl glutamine) were identified in metabolic profiles of mild asthmatics compared to healthy controls [179]. In addition, lipidomic analysis found lipid metabolism dysregulation in moderate asthma positively correlated with asthma severity, but not in mild asthma when compared to healthy controls [183].

### 5.2. Severe Asthma

According to the GINA guidelines, severe asthma (SA) is characterized by uncontrolled or partially controlled symptoms, despite consistent demands for high-dose inhaled corticosteroids (ICSs) with an additional controller or oral corticosteroids (OCSs) [218]. Metabolic analysis in SA found significant differences of metabolites, especially in amino acid metabolism with higher levels of β-alanine [182] or lysine [190]. Moreover, a lipidomic analysis revealed that 22 metabolites are changed in SA. Several lipid mediators, including sphingolipids (sphingomyelin, ceramide, and S1P), free fatty acids, and eicosanoids (LTE_4_) are positively correlated with asthma severity [179,186]. In addition, the resistance to steroids is one of the clinical characteristics in SA [219]. In particular, distinct metabolic profiles are noted in severe asthmatics using ICSs or OCSs with decreased steroid metabolites. Moreover, linoleic acids from polyunsaturated fatty acids drive steroid refractory response and contribute to asthma severity with airway epithelial injury [220,221].

### 5.3. Obese Asthma

Obese asthma is classified as a distinct phenotype of asthma with persistent symptoms and resistance to conventional medication [222]. With the increasing of the obese population, obesity now accounts for 11% of adult asthmatics [223]. Although obesity is one of the risk factors for asthma, the mechanisms are poorly understood and needed to be clarified [224]. Several studies have suggested that the metabolic dysregulation is related to development and progress of asthma [225,226]. With omics technologies, distinct metabolic changes have been identified in obese subjects [227]. In particular, high-fat diets tend to increase the concentrations of ceramide, sphingomyelin, and S1P in multiple tissue and organs [228]. Among them, ceramide could contribute to airway hyperresponsiveness, suggesting a possible correlation with asthma severity [229]. Moreover, elevated levels of C18:0 and C:20 were found in the sera of obese asthmatics [230]. Although the exact mechanisms are not comprehended yet, clinical and experimental research have suggested the specific metabolic changes in obese asthma.

## 6. Clinical Implications and Perspectives of Various Metabolites

Due to the sensitivity to biological alterations, metabolites can serve to search for novel biomarkers and pathological mechanisms for asthma phenotypes (Table 5). The application of metabolomic analysis provides new insights into the classification of asthma phenotypes in terms of the dynamic network between genetic and environmental factors [6]. In addition, the integrated omics has been used from early diagnosis to monitoring treatment response in diseases [231]. Therefore, metabolomics may lead to a precise discrimination of asthmatic patients, but also improvement in a search for new therapeutic targets in asthma as well [232]. Furthermore, the metabolic therapy is a novel concept comprising restrictive diet control or nutritional supplements with relatively easy and safe interventions. Although metabolomics is highlighted for biomarker identification for asthma, the efficacy and safety of metabolic signatures should be validated for their practical uses in clinical courses. Based on integrative omics studies in asthma, metabolites may realize the implementation of personalized medication and expand treatment options for asthmatics in a combination with current medications.

## Figures and Tables

**Table 1:** Summary of genomic analysis in adult asthmatics.

Linkage Studies with Positional Cloning			
ADAM33	20p13		[25]
DPP10	2q14		[26]
PHF11	13q14		[27]
GPRA	7p14		[28]
HLA-G	6p21		[29]
CYFIP2	5q33		[30]
IRAK3	12q14		[31]
OPN3	1q		[32]
Candidate gene association studies			
Asthma	IL10	1q31	[37]
	IL1RN	2q14	[38]
	IL1B	2q14	[39]
	HNMT	2q22	[40]
	CTLA4	2q33	[41]
	CCR3	3p21	[42]
	CCR5	3p21	[43,44]
	TLR9	3p21.3	[45]
	MUC7	4q13-21	[46]
	PGDS	4q21-22	[47]
	CSF2	5q31	[37]
	IL4	5q31	[48,49]
	IL13	5q31	[50,51,52]
	UGRP1	5q31-34	[53]
	ADRB2	5q32-34	[54,55]
	LTC4S	5q35	[56,57]
	HLA-DRB1	6p21	[58,59,60,61]
	HLA-DPB1	6p21	[62]
	TNF	6p21	[58,63,64,65,66]
	LTA	6p21	[58,65]
	TAP1	6p21	[67,68]
	PAFAH	6p21	[69,70,71]
	EDN1	6p21	[72]
	EOTAXIN2	7q11	[73]
	CFTR	7q31	[74,75,76,77]
	NOS3	7q36	[37,78]
	C5	9q34	[37]
	SDF1	10q11	[37]
	CC16/CC10	11q12-13	[79,80,81]
	FCER1B	11q12-13	[82,83,84,85,86,87,88]
	GSTP1	11q13	[89]
	AICDA	12p13	[90]
	STAT6	12q13	[91]
	NOS1	12q24	[92,93]
	ACT	14q32	[94]
	IL4RA	16p12	[95,96,97,98,99]
	RANTES	17q11-12	[100,101]
	ACE	17q23	[102,103]
	TBXA2R	19p13	[104]
Asthma severity	CTLA4	2q33	[105]
	IL4	5q31	[106]
	ADRB2	5q32-34	[107]
	PAFAH	6p21	[69]
	IL4RA	16p12	[97]
	TGFB1	19q13	[108]
Eosinophil counts	IL10	1q31	[72]
	STAT6	12q13	[109]
	NOS1	12q24	[72]
	EOTAXIN1	17q21	[110]
Total IgE in general population	CTLA4	2q33	[41]
	IL4	5q31	[111]
	CD14	5q31	[112,113]
	HLA-DRB1	6p21	[114,115,116]
	PAFAH	6p21	[70]
	IFNGR1	6q23	[117]
	FCER1B	11q13	[84]
	AICDA	12p13	[90]
	IFNG	12q21	[118]
	NOS1	12q24	[72]
	IL4RA	16p12	[95,99,119,120]
	TGFB1	19q13	[121]
	IFNGR2	21q22	[117]
	IL13RA1	Xq13	[51]
Total IgE in asthmatics	IL10	1q31	[121,122]
	CTLA4	2q33	[123]
	IL4	5q31	[124]
	NOS3	7q36	[125]
	FCER1B	11q13	[87]
	EOTAXIN1	17q21	[73]
Specific IgE	IL4	5q31	[126]
	HLA-DPB1	6p21	[127,128]
	PAFAH	6p21	[70]
	NOS3	7q36	[125]
	FCER1B	11q13	[129]
AHR/BHR	IL10	1q31	[37]
	CTLA4	2q33	[41,105]
	CSF2	5q31	[37]
	IL13	5q31	[50]
	ADRB2	5q32-34	[130,131]
	LTC4S	5q35	[37]
	TNF	6p21	[37,132,133]
	LTA	6p21	[132]
	NOS3	7q36	[37]
	FCER1B	11q13	[129]
	ACT	14q32	[94]
	IL4RA	16p12	[95]
	NOS2A	17cen-q11	[37]
FEV1/FVC	IL4	5q31	[134]
	ADRB2	5q32-34	[135]
	LTC4S	5q35	[136]
	IL4RA	16p12	[106]
	EOTAXIN1	17q21	[110]
Genome-wide association studies			
CRCT1	1q21		[137]
IL6R	1q21		[137,138]
PYHIN1	1q23		[35,137]
DENND1B/CRB1	1q31		[137]
IL18R1/IL1RL1/IL1RL2	2q12		[34,35,137,138,139]
DPP10	2q12.3-q14.2		[140]
D2HGDH	2q37		[137]
USP38/GAB1	4q31		[137,141]
PDE4D	5q12		[142]
TSLP/SLC22A5	5q22		[34,137,139,141,143]
RAD50/NDFIP1	5q31		[137,144]
ADRA1B	5q33		[140]
IL13	5q		[34,144]
HLA-DP/DQ/DR	6p21		[34,137,143,144,145,146]
BTNL2	6p21		[137]
CDHR3	8q24		[137]
TEK	9p21		[143]
IL-33	9p24		[34,138]
PTGES	9q34		[143]
GATA3	10p14		[141]
JMJDIC/REEP3	10q21		[143]
CDK2/IKZF4	12q13		[137]
RORA	15q22		[34,35,137]
SMAD3	15q22		[34,137]
ORMDL3/GSMDB/IKZF3	17q21		[34,35,137,138,139]
PRNP	20pter-p12		[140]
IL2RB	22q12.3		[34,35,137,138]

ADAM33, a disintegrin and metalloproteinase 33; DPP10, dipeptidyl peptidase 10; PHF11, PHD finger protein 11; GPRA, G-protein-coupled receptor for asthma susceptibility; HLA-G, human leukocyte antigen-G; CYFIP2, cytoplasmic FMR1-interacting protein 2; IRAK3, IL-1 receptor-associated kinase 3; OPN3, opsin 3; AHR, airway hyperresponsiveness; BHR, bronchial hyperresponsiveness; FEV1, forced expiratory volume; FVC, forced vital capacity; IL, interleukin; HNMT, histamine N-methyltransferase; CTLA4, cytotoxic T lymphocyte antigen 4; CCR, CC chemokine receptor; TLR, toll-like receptor; MUC, mucin; PGDS, prostaglandin D synthase; CSF2, colony stimulating factor 2; UGRP1, uterus globulin associated protein 1; ADRB2, adrenoceptor beta 2; LTC4S, leukotriene C4 synthase; TNF, tumor necrosis factor; LTA, lymphotoxin alpha; TAP1, antigen peptide transporter 1; PAFAH, platelet activating factor acetylhydrolase; EDN1, endothelin 1; CFTR, cystic fibrosis transmembrane conductance regulator; NOS, nitric oxide synthase; SDF1, stromal cell-derived factor 1; CC, clara cell secretory protein; FCER1B, high affinity immunoglobulin epsilon receptor subunit beta; GSTP1, glutathione S-transferase Pi 1; AICDA, activation induced cytidine deaminase; STAT6, signal transducer and activator of transcription 6; ACT, alpha-1-antichymotrypsin; IL4RA, interleukin 4 receptor alpha; RANTES, regulated on activation, normal T cell expressed and secreted; ACE, angiotensin converting enzyme; TBXA2R, thromboxane A2 receptor; TGFB1, transforming growth factor beta 1; IFNGR, interferon gamma receptor 1; IFNG, interferon gamma; IL13RA1, interleukin 13 receptor alpha 1; CRCT1; cysteine-rich C-terminal 1; IL6R, interleukin 6 receptor; PYHIN1, pyrin, and HIN domain family member 1; D2NND1B, DENN domain containing 1B; CRB1, crumbs cell polarity complex component 1; IL18R1, interleukin 18 receptor 1; IL1RL, interleukin 1 receptor like; D2HGDH, D-2-hydroxyglutarate dehydrogenase; USP38, ubiquitin specific peptidase 38; GAB1, growth factor receptor bound protein 2-associated binding protein 1; PDE4D, phosphodiesterase 4D; TSLP, thymic stromal lymphopoietin; SLC22A5, solute carrier family 22 member 5; RAD50, RAD50 double strand break repair protein; NDFIP1, Nedd4 family interacting protein 1; ADRA1B, adrenoceptor alpha 1B; HLA, major histocompatibility complex; BTNL2, butyrophilin like 2; CDHR3, cadherin related family member 3; TEK, TEK receptor tyrosine kinase; PTGES, prostaglandin E synthase; GATA3, GATA binding protein 3; JMJD1C, jumonji domain containing 1C; REEP3, receptor accessory protein 3; CDK2, cyclin dependent kinase 2; IKZF, IKAROS family zinc finger; RORA, RAR related orphan receptor A; SMAD3, mothers against decapentaplegic homolog 3; ORMDL3, ORMDL sphingolipid biosynthesis regulator 3; GSMDB, gasdermin B; PRNP, prion protein; IL2RB, interleukin 2 receptor subunit beta.

**Table 2:** Summary of transcriptomic analyses in adult asthmatics.

Population	Methods	Purpose	Findings	Ref.
Blood				
Exacerbation sample in adult asthmatics (*n* = 118)	Microarray and qPCR with pathway analysis	To identify the exacerbation-associated gene expression patterns in PBMC	TLR activation pathway with elevations in type 1 interferon and IL-15 genes is associated with asthma exacerbation.	[149]
Nonsmoking SA/smoking SA/nonsmoking mild to moderate asthma/nonsmoking controls (*n* = 246/88/77/87)	Microarray with WGCNA	To find the transcriptional differences between subgroups of asthmatics and non-asthmatics in whole blood	Differentially expressed genes in immune cells of severe asthmatics. Gene sets related to chemotaxis, mobilization, migration, and infiltration of myeloid cells contribute to asthma severity.	[150]
Airway epithelial cell				
nonsmoking asthma/nonsmoking HCs/smoking controls (*n* = 42/28/16)	Microarray and qPCR	To explore the distinct gene expression related to airway dysfunction and corticosteroid treatment in epithelial cells of asthmatics	Identification of 22 differentially expressed genes in asthmatics. IL-13-derived asthma-associated genes (CLCA1, periostin, and serpinB2) are decreased by corticosteroid treatment with an improvement in lung function.	[151]
Asthmatics/HCs (*n* = 42/28)	Microarray and qPCR	To define the molecular phenotypes based on type 2 cytokines-induced gene expression in epithelial brushings of asthmatics	Phenotyping of asthma as Th2-high and Th2-low based on CLCA1, periostin, and sepinB2 gene expression. Th2-high asthma has worse clinical outcomes (lower lung function, higher serum IgE, and blood/airway eosinophilia) and inflammatory features (subepithelial fibrosis and increase of mucin stores).	[152]
Nonsmoking SA/smoking SA/mild-to-moderate asthma/HCs (*n* = 46/16/34/41)	Microarray	To investigate the pathogenesis of severe asthma and the influences of blood, sputum, and submucosal eosinophils or neutrophils on the gene expression in bronchial brushing	7 genes (COX-2, ADAM-7, SLCO1A2, TMEFF2, TRPM-1, and 2 unnamed genes) are positively correlated with blood and submucosal eosinophilia with thick lamina reticularis and elevated F_E_NO.	[153]
SA/moderate asthma with ICS/mild asthma with ICS/mild-to-moderate asthma with no ICS/HCs (*n* = 51/19/22/37/26)	Microarray with WGCNA, LIMMA, and pathway analysis	To identify specific genetic networks to be associated with asthma severity	Asthma severity has a positive correlation with a network related to mitosis/cell division and T2 inflammation, but a negative correlation with a network related to epithelial growth/repair, cell integrity/remodeling, and neuronal function/development.	[154]
Sputum				
Asthmatics/HCs (*n* = 59/13)	Microarray and qPCR with GO and pathway analysis	To establish 3 distinct transcriptional asthma phenotypes (TAPs) considering clinical status and gene expression in the sputum of asthmatics	Classification of asthma phenotypes as eosinophilic, neutrophilic, or paucigranulocytic asthma based on the predominance of immune cells in sputum. IL-1 and TNF-α/NF-κB pathways are involved in the pathogenesis of neutrophilic asthma.	[155]
Asthmatics/HCs (*n* = 37/15)	qPCR	To determine genetic profiling related to Th2 cytokines in the sputum cells of asthmatics for the categorization of asthma phenotypes	Standardization of IL-4, IL-5, and IL-13 gene expression for classification as Th2-high and Th2-low subtypes (Th2 gene mean). Th2-high asthma has poor clinical outcomes (low lung function and blood eosinophilia) with elevation in mast cell/eosinophil-related genes.	[156]
Asthmatics/HCs (*n* = 106/20)	Microarray and qPCR	To validate genetic biomarkers for inflammatory phenotypes of asthma and prediction of ICS treatment response	Identification of 23 differentially expressed genes across eosinophilic, neutrophilic, and paucigranulocytic phenotypes. The 3 genes for eosinophilic asthma (CLC, CPA3, and DNASE1L3) and 3 for neutrophilic asthma (IL1B, ALPL, and CXCR2) are validated with distinct alterations after ICS treatments.	[157]
Asthmatics/HCs (*n* = 84/27)	RNA seq with WGCNA and pathway analysis	To identify genetic networks in sputum immune cells of asthmatics for clustering them into T2-high and T2-low subgroups	High T2-network gene expression in the T2-high asthma comes from the interaction of various immune cells (eosinophils, mast cells, basophils, and dendritic cells), leading to severe airway dysfunction. CD8+T cell network gene expression is lower in T2-low asthma and negatively correlated with body mass index.	[158]
Elderly asthmatics/HCs (*n* = 55/10)	Microarray with GSEA and cluster analysis	To find distinct biological mechanisms with genetic profiling in sputum cells for clustering of elderly asthmatics	Identification of 2 molecular clusters in elderly asthmatics with increased OXPHOS and EMT gene sets, respectively. The OXPHOS/UPR system related to oxidative stress leads to inflammatory response and immune function dysregulation in the airways of elderly asthmatics. The EMT gene sets contribute to airway remodeling with lower lung function in elderly asthmatics.	[159]
Nasal brushings				
Mild-to-moderate asthmatics/HCs (*n* = 66/124)	Microarray and RNA seq with pathway and classification analysis	To validate nasal brush-based classifier genes for the diagnosis of asthma	Identification of 90 genes as a nasal classifier for mild-to-moderate asthma	[160]
BALF				
SA/moderate asthma with ICS/mild asthma with ICS/mild-to-moderate asthma with no ICS/HCs (*n* = 44/15/18/40/37)	LIMMA, WGCNA, GO and pathway analysis	To determine severity-related genes and influence of β-agonist use on gene expression in BAL immune cells	Higher BAL neutrophils with increased gene expression related to TNF-α and type 1 interferon pathway in SA. Several severity-related genes are within or close to asthma susceptibility loci (5q, 17q, 1p). A specific gene network related to cAMP signaling is associated with asthma severity and β-agonist exposure.	[161]
Mixed				
Adult/childhood-onset SA (*n* = 253/158)	Microarray with GSVA	To identify gene signatures in adult-onset compared to childhood-onset SA using diverse samples (nasal brushings, bronchial brushings, and sputum)	Identification of 5 differentially expressed gene signatures in nasal brushings, 6 in bronchial brushings, and 3 in sputum. Specific genes related to immune cells (eosinophils, mast cell, ILC3) and type 2 inflammation are up-regulated in adult-onset SA.	[162]

HCs, healthy controls; SA, severe asthma; ICS, inhaled corticosteroid; qPCR; quantitative PCR; WGCNA, weighted gene co-expression network analysis; LIMMA, linear models for microarray data; GO, gene ontology; GSCA, gene set co-expression analysis; PBMC, peripheral blood mononuclear cell; BALF, bronchoalveolar lavage fluid; TLR, toll-like receptor; CLCA1, chloride accessory 1; COX-2, cyclooxygenease-2; ADAM-7, a disintegrin and metalloproteinase domain-containing protein 7; SLCO1A2, solute carrier organic anion transporter family member 1A2; TMEFF2, transmembrane protein with epidermal growth factor like and two follistatin like domains 2; TRPM-1, transient receptor potential cation channel subfamily M member 1; CLC. Charcot-leydon crystal protein; CPA3, carboxypeptidase A3; DNASE1L3, deoxyribonuclease 1-like 3; ALPL, alkaline phosphatase, tissue-nonspecific isozyme; CXCR2, chemokine (C-X-C motif) receptor 2; OXPHOS, oxidative phosphorylation; UPR, unfolded protein response; EMT, epithelial-mesenchymal transition; cAMP, cyclic adenosine 3′,5′-monophosphate; ILC3, innate lymphoid cell 3.

**Table 3:** Summary of proteomic analyses in adult asthmatics.

Population	Methods	Purpose	Findings	Ref.
Serum				
AIA/ATA/HCs (*n* = 30/24/21)	2D-PAGE, MALDI-TOF MS, and ELISA	To investigate differentially expressed proteins in AIA	Identification of distinct protein expression in AIA as complement components, modified albumin, apolipoprotein, PRO2619, hypothetical protein, and SPOCD1 protein. C3a and C4a levels are higher in AIA and correlated with FEV_1_, suggesting the pathogenic role of complements in AIA.	[165]
Sputum				
Asthmatics with EIB/those without EIB/HCs (*n* = 5/5/5)	LC-MS/MS with GO and network analysis	To determine the contribution of specific proteins to asthma and susceptibility for EIB	10 up-regulated (SERPINA1) and 7 down-regulated proteins (S100A9, S100A8, SMR3B, and SCGB1A1) in asthmatics are related to defense response, inflammation, and protease inhibitory activity. 9 proteins including C3 and HPX are susceptible to EIB in asthmatics.	[166]
UA/PC/CA/COPD/HCs (*n* = 20/35/21/21/8)	2D-PAGE, MALDI-TOF MS, and ELISA	To identify biomarkers for severe UA with neutrophilic inflammation	6 increased and 7 decreased proteins in severe uncontrolled asthma with neutrophilic inflammation are related to inflammatory/immunity/enzyme activity, cysteine protease inhibitor, signaling, and cytoskeleton functions. S100A9 is suggested as a UA biomarker contributing to neutrophilic airway inflammation and steroid resistance.	[167]
BALF				
Asthmatics/HCs (*n* = 4/3)	SDS-PAGE, nano-HPLC-MS/MS, and ELISA with GO analysis	To verify protein expression changes before and after segmental allergen challenge in asthmatics	Alterations in protein expression related to diverse biological functions after segmental allergen challenge in asthmatics. Several signature proteins released from immune cells (CLC, MBP, EDN, ECP, CRISP-3, and MMP-9) are elevated after challenge. Differentially expressed proteins are involved in various molecular functions (hydrolase activity, protein binding, and calcium ion binding) and biological process (immune response, lipid metabolism, transport, and signal transduction).	[168]
Atopic asthmatics/HCs (*n* = 6/6)	2D-PAGE, MALDI-TOF MS, and ELISA with functional analysis	To demonstrate the influences of IL-4 stimulation on gelsolin secretion in BALF of asthmatics	Higher concentrations of IL-4 and gelsolin in BALF of asthmatics. IL-4 treatment induces gelsolin expression in airway epithelial cells of asthmatics for mucus viscosity control and innate antimicrobial activity.	[169]
Asthmatics/HCs (*n* = 11/19)	2D-PAGE, SDS-PAGE, and MS/MS	To investigate the oxidative mechanisms in asthmatic airways and allergen-induced mouse models	Lower activity of catalase function in asthmatics contributes to increased oxidative stress, driving chronic and severe airway inflammation.	[170]
Mild asthmatics/HCs (*n* = 4/4)	Affinity chromatography and ESI LC-MS/MS with functional analysis	To identify the function of galectins in asthmatic airways	Differential airway localization of galectin-3 in epithelium, endothelium, smooth muscle cells, and fibroblasts, as well as galectin-8 in plasma cells. Distinct profiles of galectin-bound proteins in asthmatics suggest its linkage with eosinophilic inflammation and airway remodeling.	[171]
Bronchial biopsy				
Asthmatics/HCs (*n* = 12/3)	iTRAQ LC-MS/MS with functional and pathway analysis	To determine distinct proteins and related biological pathways related to asthmatics and their alterations by ICS treatment.	Identification of 7 significantly different proteins between asthmatics and HCs related to multiple biological functions (hematological system development/function, lipid metabolism, molecular transport, signaling, and tissue development). ICS treatment alters protein expression related to immune cell trafficking, tissue development, and hematological systems development/function.	[172]

AIA, aspirin-induced asthma; ATA, aspirin-tolerant asthma; HCs, healthy controls; EIB, exercise-induced bronchoconstriction; UA, uncontrolled asthma; PC, partially controlled asthma; CA, controlled asthma; COPD, chronic obstructive pulmonary diseases; 2D-PAGE, two-dimensional gel electrophoresis; MALDI, matrix-assisted laser desorption and ionization; TOF, time-of-flight; MS, mass spectrometry; LC, liquid chromatography; HPLC, high-performance liquid chromatography; ESI, electrospray ionization; iTRAQ, isobaric tag for relative and absolute quantitation; SPOCD1, spen paralogue and orthologue C-terminal domain containing 1; SERPINA1, serpin peptidase inhibitor; S100A9, S100 calcium binding protein A9; S100A8, S100 calcium-binding protein A8; SMR3B, submaxillary gland androgen-regulated protein 3B; SCGB1A1, secretoglobin; HPX, hemopexin; CLC, charcot-leyden crystal protein; MBP, major basic protein; EDN, eosinophil-derived neurotoxin; ECP, eosinophil cationic protein; CRISP-3, cysteine-rich secretory proteins; MMP-9, matrix metalloproteinase-9.

**Table 4:** Summary of metabolomic analyses in adult asthmatics.

Population	Methods	Purpose	Findings	Ref.
Blood				
Asthmatics/HCs (*n* = 147/2778)	ESI-MS/MS	To investigate lipid metabolic biomarkers associated with asthma candidate genes by genomic and metabolomic analysis in the sera of asthmatics	151 different metabolites between asthmatics and HCs. 2 significant metabolites (PC.ae.C42:1 and PC.ae.C42:5) are associated with current asthma. 6 SNPs exert effects on the production of asthma-associated metabolites: 2 SNPs at 17q21 (PSMD3, MED24) on PC.ae.C42:4, PC.ae.C42:5, PC.ae.C44:5, PC.ae.C44:6, and 1 SNP at TSLP gene on PC.aa.C34:4, SM.C20:2 and IL1RL1 gene on SM.C20:2.	[176]
Asthmatics/HCs (*n* = 39/26)	^1^H-NMR	To demonstrate distinct metabolic profiling in the sera of asthmatics and identify the potential biomarkers for asthma	10 significant metabolites in asthmatics: increase of methionine and glutamine and decrease of formate, choline, histidine, acetate, glucose, phosphocholine, arginine, and methanol. FEV_1_ has a positive correlation with choline and arginine, but a negative correlation with acetone.	[177]
Asthmatics/HCs (*n* = 17/17)	GC-TOF-MS	To explore metabolic changes and related mechanisms in the sera of mild asthmatics for novel prognostic markers	14 significant metabolites in asthmatics and 8 potential clinical indicators for asthma: succinic acid, 3,4-dihydroxybenzoic acid, inosine, 5-aminovaleric acid, phenylalanine, ascorbate, dehydroascorbic acid, and 2-ketovateric acid. Specific metabolic pathways (TCA cycle, hypoxia metabolism, and urea cycle) are associated with asthma.	[178]
Mild/moderate/severe asthmatics/HCs (*n* = 12/20/22/22)	LC-HRMS, MS/MS	To determine distinct serum metabolic profiles related to asthma severity and steroid treatment	15 significant metabolites across mild/moderate/severe asthma and HCs. 6 significant metabolites are correlated with ICS doses: DHEA-S, cortisone, ProHyp, pipecolate, N-palmitoyltaurine, and cortisol. 2 primary metabolic clusters are correlated with asthma severity: decrease of DHEA-S and increase of OEA, S1P, N-palmitoltaurine, 22-hydroxycholesterol, and xanthine. Targeted analysis for lipid metabolites reveals increases in specific ceramides, sphingomyelins, eicosanoids, and fatty acids and their correlation with asthma severity and ICS doses.	[179]
EA/NEA/HCs (*n* = 13/16/15)	UPLC-MS/MS	To identify differential metabolic patterns and pathways in specific clinical inflammatory phenotypes	18 potential metabolites for diagnostic biomarkers for EA and NEA. 3 significant metabolic pathways related to glycerophospholipid, retinol, and sphingolipid for asthma pathogenesis.	[180]
Allergic asthma/HCs (*n* = 32/50)	GC-NICI-MS	To investigate the role of prostaglandin D_2_ and its metabolites in the early asthmatic response to allergens	Targeted analysis in plasma shows early increase in 9α,11β-Prostaglandin F_2_ after allergen challenge induced by mast cell activation.	[181]
NSA/SA/HCs (*n* = 10/10/10)	UHLPC-MS/MS, GC/MS	To determine the involvement of biochemical metabolism in asthma and its severity	25 significant metabolites for asthmatics mostly related with lipid metabolism, 16 for severe asthma with steroid/amino acid metabolism, and 13 for high F_E_NO with amino acid/lipid/bile acid metabolism. Increased taurine, nicotinamide, AMP, and arachidonate in asthmatics. Decreased 1-steraroyylglycerol, degydroisoandrosterone sulfate, and androsterone sulfate in severe asthma. Contribution of valine, isoleucine, and ornithine to high F_E_NO in asthmatics.	[182]
Asthmatics/HCs (*n* = 35/32)	UHPLC-MS/MS	To identify the role of lipid metabolism for asthma and IgE levels by lipidomic analysis in plasma	10 lipid species for asthma diagnosis: PE(38:1), PE(18:1p/22:6), PE(20:0/18:1), SM(d18:1/18:1), TG(17:0/18:1/18:1), TG(16:0/16:0/18:1), PI(16:0/20:4), PG(44:9), Cer(d16:0/27:2), and LPC(22:4). Correlation of PE(20:0/18:1) and TG(16:0/16:0/18:1) IgE levels in asthmatics.	[183]
Urine				
Asthmatics (*n* = 10)	GC×GC-TOF-MS H-NMR	To identify the urinary metabolic changes related to asthma exacerbation	Contribution of lipid peroxidation to asthma exacerbation with an increase of alkanes and aldehydes in urine. Threonine, alanine, carnitine acetylcarnitine, and trimethylamine-N-oxide are increased; acetate, citrate, malonate, Hippurate, dimethylglycine, and phenylacetylglutamine are decreased in exacerbated condition.	[185]
Asthmatics (*n* = 57)	GC×GC-TOF-MS	To demonstrate the influences of metabolites related to lipid peroxidation on asthma and its clinical parameters	Increase of urine metabolites related to lipid peroxidation in asthma related to clinical characteristics (asthma severity scores, FEV_1_, F_E_NO, blood eosinophils, and serum IgE).	[186]
Smoking SA/nonsmoking SA/nonsmoking mild-to-moderate asthma/HCs (*n* = 109/302/86/100)	MS	To investigate urinary eicosanoid metabolism in asthma for its phenotyping and association with asthma therapeutic agents	Higher concentration of urinary metabolites related to PGD_2_, PGF_2__α_, PGE_2_, TXA_2_, isoprostanes, and CysLTs pathway in SA. Lower concentration of 2,3-dinor-TXB_2_ and 8,12-iso-iPF_2α_-VI in the OCS-treated group and 2,3-dinor-11β-PGF_2α_, LTE_4_, and 11-dehydro-TXB_2_ in the omalizumab-treated group. Correlation of urinary LTE_4_ with T2 inflammation markers in asthma (low lung function, blood/sputum eosinophils, and serum IgE/periostin/IL-13).	[187]
Exhaled breath condensate (EBC)				
Asthmatics/HCs (*n* = 82/35)	^1^H-NMR	To determine EBC metabolomic profiles for phenotype comparisons and ICS use	5 distinct spectral regions for asthmatics (0.16-0.18, 0.78-0.84, 0.88-0.94, 7.36-7.42, and 7.44-7.52 ppm) 2 significant spectra regions for sputum eosinophilia, 7 for sputum neutrophilia, 1 for asthma control, and 8 for steroid use.	[189]
SA/mild-to-moderate asthma (*n* = 15/21)	UHPLC-ESI-MS ^1^H-NMR	To validate discriminating metabolites of SA from mild-to-moderate asthma	Contribution of amino acid (lysine) and lipid metabolism (eicosanoids, phospholipids, and unsaturated fatty acids) to asthma severity.	[190]
Mild asthma/HCs (*n* = 55/55)	NMR	To identify differential NMR profiles of asthma in different operating temperature (−27.3 and −4.8 °C) for standardization of EBC collection.	Separation of asthmatics at −27.3 °C from HCs at −4.8 °C by uracil, urocanic acid, succinate, SFA, Phe, hippurate, trimethylamine, Val, and Tyr; from HCs at −27.3 °C by propionate, 4OH-phenylacetate, Val, acetate, SFA, Pro, Tyr, Arg, trans-aconitate, and Phe. Separation of asthmatics at −4.8 °C from HCs at −27.3 °C by Phe, succinate, Val, propionate, SFA, methanol, uracil, Pro, formate, isobutyrate, and urocanic acid; from HCs at −4.8 °C by SFA, Val, adenosine, Hippurate, Ala, formate, urocanic acid, Pro, acetate, ethanol, methanol, and Ile.	[191]
Asthmatics/HCs (*n* = 89/20)	NMR	To determine distinct metabolic patterns of asthma and its endotypes	NMR spectra region at 7 ppm for distinguishing asthmatics from HCs by up-regulation of isopropanol and N,N, dimethylglycine and by down-regulation of ammonia. Classifying asthmatics as 3 clusters characterized (1) by low exacerbation ratio, (2) by high exhaled nitric oxide, and (3) by low blood eosinophils but high blood neutrophils. Contribution of high acetate, acetone, formic acid, methanol, and N,N, dimethylglycine concentrations as well as low concentrations of ammonia and hydroxybutyrate to asthma severity.	[192]
OA/LA/HCs (*n* = 25/30/30/25)	^1^H-NMR	To investigate metabolic profiles and pathways for class-specific metabolic types	Confirmation of carbohydrate signals (3.9-3.2 ppm) in OA. 23 and 17 metabolic pathways for OA distinct from HCs and LA, respectively, mostly related to energy metabolism (methane) and carbohydrate metabolism (glyoxylate/dicarboxylate and pyruvate). Differential metabolic profiling in OA: increases in glucose, butyrate, and acetoin and decreases in formate, tyrosine, ethanol, ethylene glycol, methanol, n-valerate, acetate, SFA, and propionate compared to HCs; increases in glucose, n-valerate, acetoin, isovalerate, and 1,2-propanediol and decreases in formate, ethnol, methanol, acetone, propionate, acetate, lactate, and SFA compared to LA.	[193]
Mixed				
OA/LA (*n* = 11/22)	GC-TOF-MS	To identify obesity-associated metabolites for obese asthma in sputum and blood	11 metabolic signatures in sputum for OA related to xanthine, gluconic lactone, shikimic acid, indole-3-acetate, L-glutamic acid, 4-aminobutyric acid, benzoate, and phytosphingosine pathways. Metabolic signatures in serum of OA: increase of valine, uric acid, N-Methy-DL-alanine, and β-glycerophosphoric acid as well as decrease of asparagine 1, and d-glyceric acid. 3 metabolic signatures in PBMCs of OA: increases in 3-ydroxynorvaline 2 and decreases in 3-hydroxybutyric acid, linolenic acid, and isoleucine.	[184]

HCs, healthy controls; EA, eosinophilic asthma; NEA, non-eosinophilic asthma; OA, obese asthma; LA, lean asthma; MMA, mild-to-moderate asthma; MS, mass spectrometry; H-NMR, proton nuclear magnetic resonance; TOF, time-of-flight; HRMS, high resolution mass spectrometry; UPLC, ultra performance liquid chromatography; UHLPC, ultra high performance liquid chromatography; NICI, negative ion chemical ionization; SNP, single nucleotide polymorphism; PC.ae, acyl-alkyl-phosphatidylcholine; PC.aa, diacyl-phosphatidylcholine; SM, sphingomyelin; PSMD3, proteasome 26S subunit, non-ATPase 3; MED24, mediator complex subunit 24; TSLP, thymic stromal lymphopoietin; IL1RL1, interleukin-1 receptor-like 1; TCA, tricarboxylic acid; ICS, inhaled corticosteroid; DHEA-S, dehydroepiandrosterone sulfate; ProHyp, prolylhydroxyproline; S1P, sphinosine-1-phosphate; AMP, adenosine 5′-monohosphate; PE, phosphatidylethanolamine; SM, sphingomyelin; TG, triglyceride; PI, phosphatidylinositol; PG, phosphatidylglycerol; Cer, ceramide; LPC, lysophosphatidylcholine; PGD_2_, prostaglandin D_2_; PGF_2α_, prostaglandin F_2α_; PGE_2_, prostaglandin E_2_; CysLT; cysteinyl leukotriene; LTE_4_, leukotriene E_4_; TXA_2_, thromboxane A_2_; TXB_2_; thromboxane B_2_; SFA, saturated fatty acids; PBMC, peripheral blood mononuclear cells.

**Table 5:** Metabolic signatures for asthma and its phenotypes.

Significant Metabolic Signatures		Population or Phenotype	Sample	AUC Values	Ref.
Carbohydrates					
Glucose		A/H	serum, ↓		[177]
		O > L/ON	EBC		[193]
Monosaccharide		N > E > H	serum		[180]
Maltose		A/H	plasma, ↑		[182]
Maltotriose		A/H	plasma, ↑		[182]
D-Glyceric acid		O < L	serum		[184]
D-Glucoheptose 1		O > L	sputum		[184]
Amino acid Metabolism					
Alanine		A/H	EBC, ↑		[191]
N-Methyl-DL-alanine		O > L	serum		[184]
Arginine		A/H	serum, ↓		[177]
		A/H	EBC, ↑		[191]
Glutamine		A/H	serum, ↑		[177]
L-glutamic acid		O > L	sputum		[184]
β-glutamic acid 1		O > L	sputum		[184]
Histidine		A/H	serum, ↓		[177]
Isoleucine		O < L	PBMCs		[184]
		A/H	EBC, ↑		[191]
Methionine		A/H	serum, ↑		[177]
Phenylalanine		A/H	EBC, ↑		[191]
Proline		A/H	EBC, ↑		[191]
Tyrosine		A/H	EBC, ↓		[191]
		O < L/ON	EBC		[193]
Valine		O > L	serum		[184]
		A/H	EBC, ↓		[191]
Taurine		A/H	plasma, ↑		[182]
Gly-pro		O > L	sputum		[184]
5-Aminobaleric acid		A/H	serum, ↑	0.948	[178]
N,N-Dimethylglycine		A/H	EBC, ↑		[192]
3-Hydroxynorvaline 2		O < L	PBMCs		[184]
Lipid metabolism					
Choline		A/H	serum, ↓		[177]
Glycerolipids	Phosphatidylcholine (20:4/16:1)	H > N > E	serum		[180]
	Phosphatidylcholine (18:1/2:0)	N > E > H	serum		[180]
	Phosphatidylcholine (16:0/18:1)	E > N > H	serum		[180]
	Acyl-alkyl-phosphatidylcholine (C42:4)	A/H	serum, ↑		[176]
	Acyl-alkyl-phosphatidylcholine (C42:5)	A/H	serum, ↑		[176]
	Acyl-alkyl-phosphatidylcholine (C44:5)	A/H	serum, ↑		[176]
	Acyl-alkyl-phosphatidylcholine (C44:6)	A/H	serum, ↑		[176]
	Phosphatidylethanolamine	A/H	plasma, ↑		[182]
	Phosphatidylethanolamine (38:1)	A/H	plasma, ↑	0.746	[183]
	Phosphatidylethanolamine (18:1p/22:6)	A/H	plasma, ↑	0.731	[183]
	Phosphatidylethanolamine (20:0/18:1)	A/H	plasma, ↑	0.710	[183]
	Phosphatidylethanolamine (18:3/14:0)	E > N > H	serum		[180]
	Phosphatidylglycerol (44:0)	A/H	plasma, ↓	0.675	[183]
	Glycerophosphorylcholine	A/H	plasma, ↑		[182]
		H > N > E	serum		[180]
	Β-glycerophosphorate	O > L	serum		[184]
	Lysophosphatidylcholine (18:1)	E > N > H	serum		[180]
	Lysophosphatidylcholine (22:4)	A/H	plasma, ↓	0.689	[183]
	Phosphatidylinositol	A/H	plasma, ↓	0.723	[183]
	Triglyceride (17:0/18:0/18:0)	A/H	plasma, ↑	0.714	[183]
	Triglyceride (16:0/16:0/18:1)	A/H	plasma, ↑	0.661	[183]
	O-phosphocholine	A/H	serum, ↓		[177]
Fatty acids	Saturated fatty acid	A/H	EBC, ↓		[191]
		O < L/ON	EBC		[193]
	Arachidonic acid	N > E > H	serum		[180]
	Arachidonate (20:4n6)	A/H	plasma, ↑		[182]
	Isobutyrate	A/H	EBC, ↑		[191]
	Linolenic acid	O < L	PBMCs		[184]
	N-palmitoyltaurine	SA	serum, ↓		[179]
	Oleamide	A/H	plasma, ↓		[182]
	n-valerate	O > L	EBC		[193]
		O < ON	EBC		[193]
	Isovalerate	O > L	EBC		[193]
Sphingolipids	Sphingosine	A/H	plasma, ↑		[182]
	Sphingosine-1-phosphate	SA	serum, ↑		[179]
	Sphingomyelin (d18:1/18:1)	A/H	plasma, ↑	0.731	[183]
	Phytosphingosine	H > N > E	serum		[180]
	Phytosphingosine 2	O > L	sputum		[184]
	Sphinganine	H > N > E	serum		[180]
	Ceramide (d16:0/27:2)	A/H	plasma, ↓	0.690	[183]
Eicosanoids	Leukotriene E_4_	SA, T2	urine, ↑		[187]
	Tetranor prostaglandin D metabolites	SA, T2	urine, ↑		[187]
	2,3-dinor-11β-PGF_2α_	SA, T2	urine, ↑		[187]
	8-iso-PGF_2α_	SA	urine, ↑		[187]
	2,3-dinor-8-iso-PGF_2α_	SA	urine, ↑		[187]
Sterol/Steroids	Androsterone sulfate	A/H	plasma, ↓		[182]
	Epiandrosterone sulfate	A/H	plasma, ↓		[182]
	Dehydroepiandrosterone sulfate	SA	serum, ↓		[179]
	Glycodeoxycholate	A/H	plasma, ↑		[182]
	Taurocholate	A/H	plasma, ↑		[182]
	Lathosterol	A/H	plasma, ↑		[182]
Retinol		H > N > E	serum		[180]
Carboxylic acid					
Acetate		A/H	serum, ↓		[180]
		A/H	EBC, ↑/↓		[180]
		O < L/ON	EBC		[180]
Butyrate		A/H	EBC, ↑		[180]
		O > ON	EBC		[180]
Formate		A/H	serum, ↓		[180]
		A/H	EBC, ↓		[180]
		O < L/ON	EBC		[180]
Propionate		A/H’	EBC, ↑		[180]
		O < L/ON	EBC		[180]
TCA cycle					
Succinic acid		A/H	serum, ↑	0.976	[191]
		A/H	EBC, ↑/↓		[191]
Nucleoside/Nucleotide					
Inosine		A/H	serum, ↑	0.962	[178]
Adenosine		A/H	EBC, ↓		[191]
Other organic compounds					
Acetoin		O > L/ON	EBC		[193]
Alcohol	Ethanol	A/H	EBC, ↑		[191]
		O < L/ON	EBC		[193]
	Ethylene glycol	O < ON	EBC		[193]
	Isopropanol	A/H	EBC, ↑		[192]
	Methanol	A/H	serum, ↓		[177]
		A/H	EBC, ↑		[177]
		A/H	EBC, ↑		[191]
		O < L/ON	EBC		[191]
	1,2-propanediol	O > L	EBC		[193]
Amines	Trimethylamine	A/H	EBC, ↓		[191]
Ascorbate		A/H	serum, ↑	0.917	[178]
3-aminopropionitrile 1		O > L	sputum		[184]
Benzene	3,4-Dihydroxybenzoic acid	A/H	serum, ↑	0.965	[178]
	Hippurate	A/H	EBC, ↓		[191]
3-Hydroxybutyric acid		O > L	sputum		[184]
Ketones bodies	Acetone	A/H	serum, ↓		[177]
		O < L	EBC		[193]
	2-Ketovaleric acid	A/H	serum, ↑	0.874	[178]
Lactate		O < L	EBC		[193]
Urocanic acid		A/H	EBC, ↓		[191]
Dehydroascorbic acid		A/H	serum, ↑	0.896	[178]
Urea		A/H	plasma, ↓		[182]
		A/H	EBC, ↑		[192]
Uric acid		O > L	serum, ↑		[184]
Xanthine		O > L	sputum		[184]
Inorganic compounds					
Ammonia		A/H	EBC, ↓		[192]
Pyrophosphate-3		O > L	sputum		[184]

A, asthmatics; H, healthy controls; O, obese asthmatics; L, lean asthmatics; ON, obese non-asthmatics; E, eosinophilic asthma; N, non-eosinophilic asthma; SA, severe asthma; T2, type 2 inflammation marker; ↓, lower in asthma; ↑, higher in asthma.
